# Insurance impacts survival for children, adolescents, and young adults with bone and soft tissue sarcomas

**DOI:** 10.1002/cam4.2739

**Published:** 2019-12-15

**Authors:** Neela L. Penumarthy, Robert E. Goldsby, Stephen C. Shiboski, Rosanna Wustrack, Patricia Murphy, Lena E. Winestone

**Affiliations:** ^1^ Division of Bioethics and Palliative Care Department of Pediatrics University of Washington School of Medicine Seattle Children's Hospital Seattle WA USA; ^2^ Division of Oncology Department of Pediatrics UCSF Benioff Children’s Hospital San Francisco CA USA; ^3^ Helen Diller Family Comprehensive Cancer Center UCSF San Francisco CA USA; ^4^ Department of Epidemiology and Biostatistics UCSF San Francisco CA USA; ^5^ Department of Orthopaedic Surgery UCSF San Francisco CA USA; ^6^ Division of Allergy, Immunology, and BMT Department of Pediatrics UCSF Benioff Children’s Hospital San Francisco CA USA

**Keywords:** AYA, cancer disparities, health insurance, pediatrics, sarcoma

## Abstract

**Background:**

While racial/ethnic survival disparities have been described in pediatric oncology, the impact of income has not been extensively explored. We analyzed how public insurance influences 5‐year overall survival (OS) in young patients with sarcomas.

**Methods:**

The University of California San Francisco Cancer Registry was used to identify patients aged 0‐39 diagnosed with bone or soft tissue sarcomas between 2000 and 2015. Low‐income patients were defined as those with no insurance or Medicaid, a means‐tested form of public insurance. Survival curves were computed using the Kaplan‐Meier method and compared using log‐rank tests and Cox models. Causal mediation was used to assess whether the association between public insurance and mortality is mediated by metastatic disease.

**Results:**

Of 1106 patients, 39% patients were classified as low‐income. Low‐income patients were more likely to be racial/ethnic minorities and to present with metastatic disease (OR 1.96, 95% CI 1.35‐2.86). Low‐income patients had significantly worse OS (61% vs 71%). Age at diagnosis and extent of disease at diagnosis were also independent predictors of OS. When stratified by extent of disease, low‐income patients consistently had significantly worse OS (localized: 78% vs 84%, regional: 64% vs 73%, metastatic: 23% vs 30%, respectively). Mediation analysis indicated that metastatic disease at diagnosis mediated 15% of the effect of public insurance on OS.

**Conclusions:**

Low‐income patients with bone and soft tissue sarcomas had decreased OS regardless of disease stage at presentation. The mechanism by which insurance status impacts survival requires additional investigation, but may be through reduced access to care.

## Graphical abstract

Low‐income patients with bone and soft tissue sarcomas had decreased overall survival compared to patients with private insurance regardless of disease stage at presentation. This survival disparity is partially mediated by presentation with advanced disease.

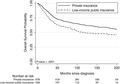

## 1 BACKGROUND

Racial and ethnic survival disparities have been described for many pediatric malignancies.^1^, ^2^, ^3^ These differences are suspected to be due, in part, to differences in tumor biology, as inferior outcomes have been demonstrated in certain ethnic groups independent of socioeconomic status (SES).^4^ However, tumor biology alone cannot explain these survival disparities. In studies of adult cancer, poverty has been shown to contribute to survival disparities through diminished access to health care, resulting in delays in diagnosis and treatment and leading to presentation with advanced disease that is more difficult to cure.^5^, ^6^, ^7^, ^8^, ^9^, ^10^ The impact of SES has not been extensively explored in the pediatric cancer setting.

While sarcomas are very rare among adult malignancies, they represent 12%‐15% of all pediatric tumors, with approximately 1700 children and young adults diagnosed yearly in the United States.^11^ They comprise a heterogeneous group of tumors, categorized according to their tissue of origin (soft tissue or bone), and are treated with a combination of surgery and/or radiotherapy to achieve local control, and chemotherapy directed toward occult or overt metastatic disease. Despite improvements in outcomes over the last four decades due to use of multimodal therapy—an approach informed by the work of multi‐institutional cooperative groups—approximately one‐third of patients, nonetheless, succumb to their disease.^12^, ^13^, ^14^

To assess whether SES affects pediatric sarcoma outcomes, we evaluated low‐income public health insurance as a proxy for income. Medicaid is a state and federally funded program which provides health insurance to low‐income patients; coverage is available under state law to eligible low‐income individuals or families.^15^, ^16^ We analyzed how low‐income public health insurance influenced overall survival (OS) in children, adolescents, and young adults diagnosed with bone and soft tissue sarcomas.

## 2 METHODS

We performed a retrospective analysis of data from the University of California San Francisco Cancer Registry. Patients were included if they were diagnosed with bone or soft tissue sarcoma between 2000 and 2015 and if they were aged between 0 and 39 years at the time of their diagnosis. We identified 1123 potentially eligible records. Of these, six were excluded because they were incorrectly categorized as sarcoma and 11 were excluded because insurance status was unknown. A total of 1106 records were available for complete analysis.

Our two primary outcomes of interest were OS and stage of disease at diagnosis. Stage of disease was coded as localized, regional, or metastatic according to American Joint Committee on Cancer (AJCC) staging assessments. The primary exposure of interest was health insurance type, defined in the University of California San Francisco Cancer Registry as the primary insurance carrier or method of payment at diagnosis.

In the UCSF Cancer Registry, data on patient demographics, primary tumor site, tumor morphology and stage at diagnosis, and follow‐up for vital status are routinely collected. The UCSF Cancer Registry classifies race based on information from the medical record. Hispanic ethnicity was determined using stated ethnicity in the medical record, national origin on the death certificate, spoken language, place of birth, and surname. For this analysis, race and ethnicity were considered a compound variable; patients were grouped into five different groups: white non‐Hispanic, white Hispanic, Asian non‐Hispanic (Asian), African American non‐Hispanic (African American), and American Indian. Bone and soft tissue sarcomas encompass a diverse array of histologic subtypes, with heterogeneity in prognosis and treatment; we performed a subgroup analysis of patients with the four most common histologic subtypes. Treatment data, including method of local control, were inconsistently collected. Thus, treatment modality variables were not included in the analysis.

### 2.1 Statistical analyses

We extracted raw data and imported into Stata 15 (Stata‐Corp, College Station, TX) for analysis. We calculated descriptive statistics for patient and tumor characteristics, and evaluated differences between groups using Chi‐square tests. We defined OS based on all‐cause mortality from diagnosis, with survival times calculated as the number of months between date of diagnosis and death. We assigned censoring times for surviving individuals using the date of last follow‐up, or 31 December 2015. We summarized OS using Kaplan‐Meier estimates^17^ with 95% confidence intervals. We evaluated between‐group differences using the log‐rank test. We generated Kaplan‐Meier plots for all patients and according to the extent of disease at presentation (localized, regional, or metastatic). The median follow‐up time for the analyzed cohort was 59 months. We truncated Kaplan‐Meier plots at 200 months. However, all available data were used in estimates of distributions and group comparisons.

We used univariate and multivariable Cox regression^18^ to evaluate predictors of OS; covariates included sex, age (<15 years, 15‐29 years, or >29 years of age), race/ethnicity, and disease stage at diagnosis. We performed subgroup analysis of patients with the four most common histologic subtypes, and adjusted for covariates listed above, as well as histology. Covariates were chosen a priori based on the literature and on the potential for confounding. We used hazard ratios (HR) and 95% confidence intervals to evaluate these covariates as predictors of OS. The proportional hazards assumption was evaluated using Schoenfeld residuals after fitting all Cox models. To evaluate whether the impact of insurance was greater among patients with more advanced disease, we performed a test of interaction on the multiplicative scale between low‐income public insurance and stage of disease at diagnosis.

To evaluate the potential impact of excluding patients missing stage of disease at diagnosis, we used chained multiple imputation techniques to fill in missing values based on modeled relationships with other analysis variables.^19^ This approach to missing data has been validated by a number of simulation studies^20^, ^21^ and has been employed in studies utilizing population‐based cancer registry data.^22^ Stage of disease at diagnosis had 17% missing data, and was the only imputed variable.

In order to evaluate the hypothesis that the association between public insurance status and mortality is mediated by the presence of metastatic disease, we used methods for causal mediation analysis.^23^ This approach is similar to the standard Baron and Kenny method for linear regression models,^24^ but is appropriate for nonlinear models for binary and survival outcomes, and incorporates a more flexible means for control of possible confounding variables.

As a prerequisite for mediation, we considered whether patients with public insurance were more likely to present with advanced stage disease. Logistic regression was used to assess the relationship between insurance and disease stage at diagnosis. Once confirmed, we included public insurance status as the primary predictor variable, and the presence of metastatic disease as the mediator, both represented as binary indicators. These variables were included in a parametric survival regression model for the OS outcome, which was assumed to follow a Weibull distribution, with surviving individuals treated as censored. The relationship between public insurance status and the presence of metastases (the mediator) was modeled with logistic regression. Both models adjusted for age at diagnosis, white (vs non‐white) race, and gender (male vs female) as possible confounding factors. Results were summarized as the estimated percentage of the effect of having public insurance on OS mediated by the presence of metastatic disease with a 95% confidence interval.

All statistical comparisons were two‐sided and significance was achieved at level *P* < .05.

## 3 RESULTS

### 3.1 Patient and tumor characteristics

The study population included 1106 patients. Insurance type was coded as uninsured (n = 8), any low‐income public insurance (Total [n = 420]: Medicaid [n = 318], Medicaid through managed care [n = 53], county funded [n = 20], Indian/public health service [n = 14], Medicare with Medicaid supplement [n = 15]), or private insurance (Total [n = 678]: HMO [n = 104], PPO [n = 136], managed care [n = 415], Tricare [n = 6], Medicare [n = 11], fee‐for‐service [n = 3], military coverage [n = 3]). Given the small number of uninsured patients, these patients were included in the low‐income public insurance group.

Of the total population, 420 patients (38.7%) had public insurance and eight patients (0.7%) had no insurance. The demographic characteristics of the study population according to insurance status are shown in Table 1. Significant differences in insurance were noted according to race/ethnicity with the public insurance group containing a higher proportion of Hispanic and African American patients. No statistically significant differences in distribution were found with regard to age, sex, or primary site. The tumor characteristics of the study population according to insurance status are shown in Table 2. A greater proportion of low‐income public insurance patients presented with regional or metastatic disease than those patients with private insurance (*P* < .001). There was a higher proportion of bone tumors in the low‐income public insurance group compared to the private insurance group (*P* = .011). The histologic subtypes that were represented and their relative proportions in the sample are shown in Table S1. The histologic subtypes with the highest representation in the sample were osteosarcoma (n = 232, 20.98%), rhabdomyosarcoma (n = 132, 11.9%), Ewing sarcoma (n = 113, 10.2%), and chondrosarcoma (n = 70, 6.3%).

**Table 1:** Demographic characteristics of cohort by insurance type

	Low‐income public insurance	Private insurance	*P*
	N = 428	N = 678	
Age (y)	21.2 (±10.75)	22.4 (±10.78)	.06
Sex			
Female	163 (38.1%)	298 (44.0%)	.075
Male	264 (61.7%)	380 (56.1%)	
Race/ethnicity			
White, non‐Hispanic	154 (36%)	455 (67.1)	<.001
White, Hispanic	188 (43.9%)	98 (14.5%)	
African American	30 (7.0%)	24 (3.5%)	
Asian/Pacific Islander	52 (12.2%)	98 (14.5%)	
American Indian	4 (0.9%)	3 (0.4%)	

**Table 2:** Tumor characteristics of cohort by insurance type

	Low‐income public insurance (N = 428)	Private insurance (N = 678)	*P*
Tissue origin			
Bone	181 (42.3%)	235 (34.7%)	.011
Soft tissue	247 (57.7%)	443 (65.3%)	
Primary site			
Non‐pelvis	362 (84.5%)	570 (84.1%)	.82
Pelvis	66 (15.4%)	108 (15.9%)	
Stage at diagnosis			
Localized	154 (36.0%)	308 (45.4%)	<.001
Regional	115 (26.9%)	164 (24.2%)	
Metastatic	91 (21.3%)	85 (12.5%)	
Unknown	68 (15.9%)	121 (17.8%)	

### 3.2 Differences in overall survival by insurance

**Figure 1:**
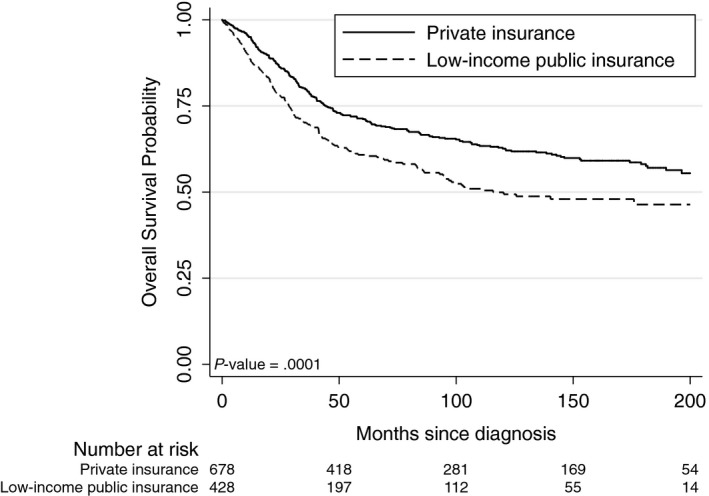
Kaplan‐Meier curves of overall survival by low‐income insurance status among patients with bone and soft tissue sarcomas

**Figure 2:**
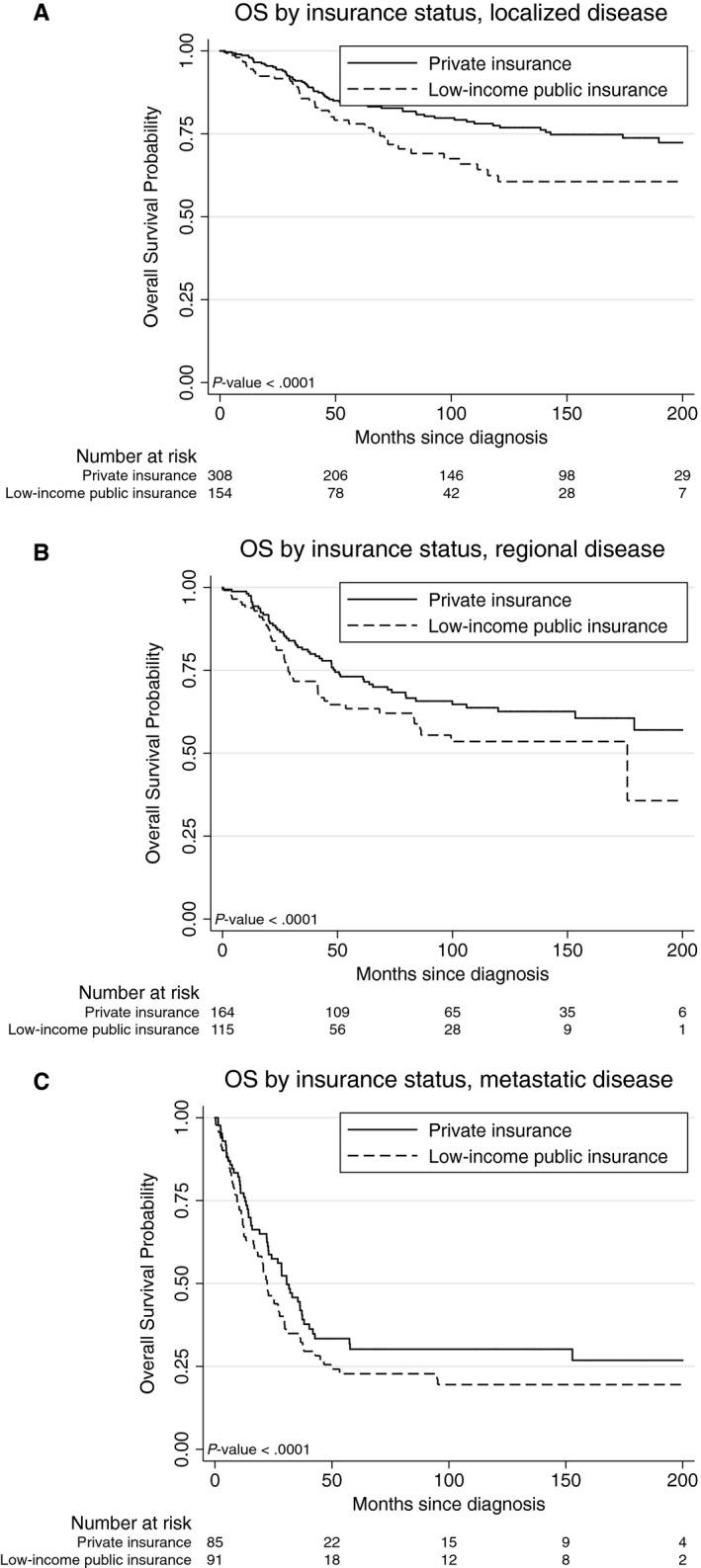
Kaplan‐Meier curves of overall survival by low‐income insurance status among patients with (A) localized, (B) regional, and (C) metastatic bone and soft tissue sarcomas

The 5‐ and 10‐year OS rates for the entire cohort were 66% and 58%, respectively. There was a statistically significant difference in OS by insurance status (HR 1.26, 95% CI 1.21‐1.78, *P* = .0001, Figure 1); patients with low‐income public insurance had significantly worse 5‐ and 10‐year OS compared to those with private insurance (61% vs 71%, 49% vs 63%, respectively, *P* = .0001). When stratified by localized, regional, or metastatic disease, those with low‐income public insurance had worse 5‐year OS compared to those with private insurance across all three strata: localized (78% vs 84%), regional (64% vs 73%), and metastatic (23% vs 30%, *P* < .0001 for each comparison). These differences in survival persisted at 10 years: localized (60% vs 77%), regional (54% vs 63%), and metastatic (19% vs 30%, *P* < .0001 for each comparison) (Figure 2). There were no significant survival differences between the bone and soft tissue sarcoma groups (HR 0.92, 95% CI: 0.76‐1.13, *P* = .44) irrespective of insurance status.

### 3.3 Univariate and multivariable analyses

Race/ethnicity was not significantly associated with OS in univariate analysis. Significant univariate predictors of OS included male sex (HR = 1.26, 95% CI: 1.04‐1.55, *P* = .021), age 15‐29 at diagnosis (HR = 1.34, 95% CI 1.06‐1.70, *P* = .015), regional disease at diagnosis (HR 1.50, 95% CI 1.15‐1.95, *P* = .002), metastatic disease at diagnosis (HR 3.95, 95% CI 3.05‐5.10, *P* < .001), and low‐income public insurance (HR 1.46, 95% CI 1.20‐1.78, *P* < .001). In multivariable analysis, the association of survival with male sex resolved when controlling for other factors. However, age 15‐29 at diagnosis (HR = 1.38, 95% CI 1.08‐1.75, *P* = .009), regional disease at diagnosis (HR 1.47, 95% CI 1.13‐1.93, *P* = .004), metastatic disease at diagnosis (HR = 3.79, 95% CI 2.90‐4.94, *P* < .001), and low‐income public insurance (HR = 1.27, 95% CI 1.02‐1.57, *P* = .032) remained significant independent predictors of OS (Table 3).

**Table 3:** Univariate and multivariable Cox proportional hazard models evaluating the hazard of survival for patients with bone and soft tissue sarcomas

	Univariate			Multivariable		
	HR	95% CI	*P*	HR	95% CI	*P*
Sex						
Female	Reference			Reference		
Male	1.26	1.04‐1.55	**.021**	1.06	0.84‐1.34	.59
Age at diagnosis						
<15	Reference			Reference		
15‐29	1.34	1.06‐1.70	**.015**	1.38	1.08‐1.75	**.009**
>29	1.00	0.78‐1.28	.982	1.09	0.85‐1.41	.50
Race/ethnicity						
NHW	Reference			Reference		
All other race/ethnicities	1.07	0.88‐1.30	.473	0.96	0.78‐1.19	.71
Stage						
Local	Reference			Reference		
Regional	1.50	1.15‐1.95	**.002**	1.47	1.13‐1.93	**.004**
Metastatic	3.95	3.05‐5.10	**<.001**	3.79	2.90‐4.94	**<.001**
Insurance status						
Private insurance	Reference			Reference		
Low‐income public insurance	1.46	1.20‐1.78	**<.001**	1.27	1.02‐1.57	**.032**

Bold text indicates a statistically significant difference with a *P* value less than .05.

In subgroup analysis of patients with the four most common histologic subtypes (osteosarcoma, rhabdomyosarcoma, Ewing sarcoma, chondrosarcoma, n = 547), histologic subtype (osteosarcoma: HR 2.91, 95% CI 1.49‐5.67, *P* = .002; rhabdomyosarcoma: HR 3.34, 95% CI 1.67‐6.69, *P* = .001), age at diagnosis (age 15‐29: HR 1.66, 95% CI 1.23‐2.23, *P* = .001; age >29: HR 2.87, 95% CI 1.82‐4.51, *P* < .001), metastatic disease at diagnosis (HR 3.8, 95% CI 2.57‐5.63, *P* < .001), and low‐income public insurance (HR 1.38, 95% CI 1.02‐1.86, *P* = .035) were significant predictors of OS in multivariable analysis (Table S2).

Patients with low‐income public insurance were more likely than those with private insurance to present with metastatic disease (OR 1.96, 95% CI 1.35‐2.86, *P* < .001) after accounting for sex, age at diagnosis, and race/ethnicity. We did not find evidence of a statistically significant interaction between having low‐income public insurance and stage of disease at diagnosis (Wald test *P* = .60). Mediation analysis indicates that metastatic disease at diagnosis mediates 15.3% (95% CI: 5%‐34%) of the association between public insurance and OS. Corresponding results incorporating imputed data for missing disease stage at diagnosis were similar.

## 4 DISCUSSION

Our analyses demonstrate that pediatric, adolescent, and young adult bone and soft tissue sarcoma patients with public insurance had inferior OS compared to patients with private insurance. This survival disparity was noted irrespective of stage of disease at diagnosis. While patients with public insurance were more likely to present with metastatic disease at diagnosis, public insurance was independently associated with diminished survival, even when controlling for sex, race/ethnicity, age, and stage of disease, as well as when stratified by stage of disease. In subgroup analysis, using the four most common histologic subtypes represented in the sample, public insurance remained independently associated with inferior survival even when controlled for histologic subtype, as well as sex, race/ethnicity, age, and stage of disease.

In this study, we used public insurance such as Medicaid as a proxy to identify low‐income patients, as coverage is only available under California state law to individuals or families whose income is near the threshold for poverty. Studies of adult cancer have implicated SES in outcome disparities in a host of cancers, particularly those where availability of screening allows for early detection such as breast and colon cancer.^25^, ^26^ Other authors have noted worse cancer outcomes in Medicaid recipients^27^, ^28^ and have theorized that while access to screening and early detection may be a contributor, the disparities are more broadly attributable to social determinants of health,^29^ such as poverty itself. Patients who are enrolled in Medicaid often face a myriad of obstacles to medical care such as lack of transportation,^30^, ^31^, ^32^ lack of social support,^33^, ^34^ lower health literacy,^35^, ^36^, ^37^ and lack of funds for deductibles and prescriptions.^38^ Additionally, Medicaid patients may be vulnerable to physiologic stressors, such food and housing insecurity^39^, ^40^, ^41^ and toxic stress, which affect their overall health status.^42^ However, our cancer registry lacks information related to individual socioeconomic characteristics such as income, education, and employment, which would assist in interrogating the association between public insurance and survival further.

Adolescents and young adults (AYA) aged 15‐29 in our study had inferior survival compared with younger children and older adults, which is consistent with the current literature.^43^, ^44^ The role of insurance in AYA cancer disparities has been explored by several authors, given the historical underinsurance of this age group.^45^ Previous studies have found that among young adults with cancer, private insurance coverage was associated with decreased likelihood of metastatic disease and death^5^, ^6^; however, patients with public insurance were handled differently in each analysis. In explaining these inferior outcomes among the AYA population, researchers have postulated that lack of insurance could result in diagnostic delays and subsequent presentation with later stage (and more difficult to treat) disease. In fact, Martin et al found that among young adults, health insurance type was significantly associated with lagtimes between onset of cancer symptoms and definitive diagnosis, and that longer lagtimes were associated with more advanced stage disease.^9^ The Affordable Care Act, which allows young adults to remain on their parents' insurance plan until the age of 26 years, has the potential to improve access to medical care in this vulnerable group.^51^ While expansion of insurance access will likely affect population health positively, concurrent measures directed toward poverty eradication would likely have synergistic benefits for cancer outcomes.

Another possible mechanism for the inferior survival in those with public insurance could relate to receipt of insufficient treatment. Specifically, achievement of local control is of paramount importance in sarcoma management; however, the optimal mode of local control (surgical resection vs radiation) remains unclear.^47^, ^48^ Previous studies have demonstrated racial disparities in receipt of surgical treatment for adult malignancies^49^, ^50^; in particular, a study of pediatric and young adult patients with chest wall sarcoma demonstrated racial disparities in receipt of surgical resection with associated inferior survival.^51^ Unfortunately, we lacked adequate data regarding treatment, such as method of local control, to ascertain whether there were treatment differences between insurance groups that could explain the noted disparities. Additionally, inferior access to care for complications of therapy and potentially increased risk of complications related to host factors (such as general nutritional status and the presence of medical comorbidities) could also influence survival.

Our data support the hypothesis that presentation with later stage disease partially mediates the relationship between low‐income public insurance and death, as there was a clear association between public insurance and advanced stage disease. Furthermore, adjustment for stage of disease decreased the hazard ratio for the relationship between public insurance and survival. However, stage only partially explains this disparity, and cannot explain the inferior survival outcomes we noted among patients with localized or regional disease. It is possible that those patients with localized and regional disease received suboptimal treatment or disease surveillance, or were unable to adhere to recommended follow‐up. In addition, our mediation analysis is vulnerable to unmeasured confounders, such as patient's primary language, education, and indicators of individual‐ and community‐level poverty, introducing bias.

While sample size and quality of curated, structured registry data are strengths of this study, several limitations should be mentioned. Within our data, the rate of uninsurance (<1% of total sample) is lower than what has been described in the literature,^46^ and may be explained by insurance enrollment at diagnosis. Moreover, insurance status is vulnerable to misclassification, as individuals may be retroactively enrolled on Medicaid at diagnosis. This misclassification may be differential if those with more advanced disease were more likely to be uninsured. This misclassification could suggest that an association exists between low‐income public insurance and metastatic disease, when truly uninsurance was the driver. Prior studies have reported that uninsured patients have distinctly inferior outcomes compared with those with public or private insurance.^5^, ^10^, ^52^ Because public insurance has been handled differently by different authors, the existing literature is difficult to extrapolate; risks associated with discrete insurance types should be queried in future studies. Additionally, the lack of reliable treatment detail in our data makes a deeper understanding of the mechanism behind the association between public insurance and survival difficult to discern.

Our findings demonstrate that public insurance is a strong independent risk factor for advanced disease at diagnosis and inferior OS among pediatric, adolescent, and young adults with bone and soft tissue sarcoma. While the survival disparity is partially mediated by advanced disease at diagnosis, other mechanisms likely explain the residual disparity. Additional studies to evaluate other mechanisms that underlie the survival disparity are essential in order to target interventions to the appropriate contributing factors. In particular, the role of poverty in pediatric cancer disparities merits further investigation.

## Data Availability

The data that support the findings of this study are available on request from the corresponding author. The data are not publicly available due to privacy or ethical restrictions.
