# Supplementary material for: Insurance impacts survival for children, adolescents, and young adults with bone and soft tissue sarcomas
Source: Cancer Med. 2019 Dec 15;9(3):951–8. doi: 10.1002/cam4.2739 (PMC6997066; doi:10.1002/cam4.2739)
Supplement: Supplementary file 1 [file CAM4-9-951-s001.docx]

**SUPPLEMENTAL TABLES**

Supplemental Table 1. Histologic subtypes of bone and soft tissue sarcoma in sample

| Histologic subtype | N (%) |  |
| --- | --- | --- |
| Adenosarcoma | 26 (2.35) | |
| Alveolar soft part sarcoma | 23 (2.08) | |
| Angiosarcoma | 12 (1.08) | |
| Carcinosarcoma | 7 (0.63) | |
| Chondrosarcoma | 70 (6.33) | |
| Clear cell sarcoma | 12 (1.08) | |
| Dermatofibrosarcoma | 35 (3.16) | |
| Desmoplastic small round cell tumor | 30 (2.71) | |
| Epithelioid | 26 (2.35) | |
| Ewing sarcoma | 113 (10.22) | |
| Fibromyxosarcoma | 18 (1.63) | |
| Fibrosarcoma | 9 (0.81) | |
| Giant cell | 22 (1.99) | |
| Hemangiosarcoma | 5 (0.45) | |
| Infantile fibrosarcoma | 7 (0.63) | |
| Leiomyosarcoma | 51 (4.61) | |
| Liposarcoma | 46 (4.16) | |
| Malignant fibrous histiocytoma | 19 (1.72) | |
| Myofibroblastic sarcoma | 9 (0.81) | |
| Myxosarcoma | 12 (1.08) | |
| NOS | 43 (3.89) | |
| Osteosarcoma | 232 (20.98) | |
| Rhabdoid tumor | 19 (1.72) | |
| Rhabdomyosarcoma | 132 (11.93) | |
| Spindle cell | 35 (3.16) | |
| Synovial sarcoma | 62 (5.61) | |
| Undifferentiated | 31 (2.80) | |

Supplemental Table 2. Univariate and multivariable Cox proportional hazard models evaluating the hazard of survival for patients with 4 most common histologic subtypes, and adjusting for histology (N=547)

|  | Univariate | | | Multivariable | | |
| --- | --- | --- | --- | --- | --- | --- |
|  | HR | 95% CI | p | HR | 95% CI | p |
| **Histology** |  |  |  |  |  |  |
| *Chondrosarcoma* | Reference |  |  | Reference |  |  |
| *Osteosarcoma* | 1.96 | 1.16-3.28 | **0.011** | 2.91 | 1.49-5.67 | **0.002** |
| *Ewing sarcoma* | 2.00 | 1.15-3.50 | **0.015** | 2.01 | 0.98-4.12 | 0.057 |
| *Rhabdomyosarcoma* | 2.84 | 1.67-4.85 | **<0.001** | 3.34 | 1.67-6.69 | **0.001** |
| **Sex** |  |  |  |  |  |  |
| *Female* | Reference |  |  | Reference |  |  |
| *Male* | 1.40 | 1.05-1.86 | **0.020** | 1.15 | 0.85-1.4 | 0.374 |
| **Age at diagnosis** |  |  |  |  |  |  |
| *<15* | Reference |  |  | Reference |  |  |
| *15-29* | 1.51 | 1.13-2.01 | **0.005** | 1.66 | 1.23-2.23 | **0.001** |
| *>29* | 1.40 | 0.94-2.09 | 0.095 | 2.87 | 1.82-4.51 | **<0.001** |
| **Race/ethnicity** |  |  |  |  |  |  |
| *NHW* | Reference |  |  | Reference |  |  |
| *All other race/ethnicities* | 1.01 | 0.77-1.31 | 0.96 | 0.82 | 0.61-1.10 | 0.177 |
| **Stage** |  |  |  |  |  |  |
| *Local* | Reference |  |  | Reference |  |  |
| *Regional* | 1.40 | 0.98-1.99 | 0.064 | 1.29 | 0.89-1.87 | 0.178 |
| *Metastatic* | 4.08 | 2.88-5.77 | **<0.001** | 3.80 | 2.57-5.63 | **<0.001** |
| **Insurance status** |  |  |  |  |  |  |
| *Private insurance* | Reference |  |  | Reference |  |  |
| *Low-income public insurance* | 1.51 | 1.16-1.97 | **0.002** | 1.38 | 1.02-1.86 | **0.035** |
